# Low-Dose Aspirin for the Primary Prevention of Cardiovascular Disease in Diabetic Individuals: A Meta-Analysis of Randomized Control Trials and Trial Sequential Analysis

**DOI:** 10.3390/jcm8050609

**Published:** 2019-05-05

**Authors:** Ming-Hsun Lin, Chien-Hsing Lee, Chin Lin, Yi-Fen Zou, Chieh-Hua Lu, Chang-Hsun Hsieh, Cho-Hao Lee

**Affiliations:** 1 Division of Endocrinology and Metabolism, Department of Internal Medicine, Tri-Service General Hospital, National Defense Medical Center, Taipei 11490, Taiwan; tim6801@msn.com (M.-H.L.); doc10383@gmail.com (C.-H.L.); undeca2001@gmail.com (C.-H.L.); 2 School of Public Health, National Defense Medical Center, Taipei 11490, Taiwan; xup6fup0629@gmail.com; 3 Department of Research and Development, National Defense Medical Center, Taipei 11490, Taiwan; 4 Department of Pharmacy, Tri-Service General Hospital, National Defense Medical Center, Taipei 11490, Taiwan; zou.yi.fen@gmail.com; 5 Department of Medical Research, National Defense Medical Center, Taipei 11490, Taiwan; 6 Division of Hematology and Oncology Medicine, Department of Internal Medicine, Tri-Service General Hospital, National Defense Medical Center, Taipei 11490, Taiwan

**Keywords:** aspirin, primary prevention, diabetes mellitus, meta-analysis, trial sequential analysis

## Abstract

Background: Evidence of low-dose aspirin as the primary prevention strategy for cardiovascular disease (CVD) in diabetes are unclear. This study was designed to evaluate the effect of low-dose aspirin use for the primary prevention of CVD in diabetes. Methods: We collected randomized controlled trials of low-dose aspirin for the primary prevention of CVD in adults with diabetes lasting at least 12 months from Medline, Embase, and the Cochrane Library up to 10 November 2018. Two reviewers extracted data and appraised the reporting quality according to a predetermined protocol (CRD4201811830). This review was conducted using Cochrane standards, trial sequential analysis, and the Grading of Recommendation. The primary outcomes were major adverse cardiovascular events (MACE, including non-fatal myocardial infarction, ischemia stroke, and cardiovascular death) and an incidence of major hemorrhage (major intracranial hemorrhage and major gastrointestinal bleeding). Results: In this primary prevention (number = 29,814 participants) meta-analysis, low-dose aspirin use reduced the risk of MACE by 9% and increased the risk of major hemorrhage by 24%. The benefits were only observed in subjects of age ≥ 60 years while reducing the same risk of MACE. In efficacy, it reduced the risk of stroke but not myocardial infarction. No increase in all-cause mortality or cardiovascular death was observed. Conclusions: We suggested the use of low-dose aspirin as the primary prevention strategy for CVD in diabetes, particularly in an older population. The absolute benefits were largely counterbalanced by the bleeding hazard.

## 1. Introduction

The leading cause of mortality and morbidity in diabetes is cardiovascular disease (CVD) [1]. Additionally, diabetes patients have a twofold increased risk of CVD (including coronary heart disease, stroke, and vascular deaths). It has been well established that aspirin shows benefits in reducing the cardiovascular morbidity and mortality as secondary prevention in subjects of diabetes [2]. Although there is no strong evidence for a specific dosage, low-dose aspirin use (81 or 100 mg/day) is common in clinical practice. Higher doses do not increase efficacy but increase the risk of bleeding [3]. However, the role of aspirin in the primary prevention of CVD in subjects of diabetes remains inconclusive [4].

The Antithrombotic Treatment Trialists’ Collaboration in 2009 demonstrated that aspirin had no significant effect in diabetic patients. However, this previous consensus was controversial because of an apparent sex and age-related bias [5]. American Diabetes Association 2019 suggested that low-dose aspirin use (75–162 mg/day) was appropriate as the primary prevention strategy in diabetic patients aged more than 50 years old or individual with one additional cardiovascular risk factor and no increased risk of major bleeding [6]. The European Guidelines on CVD prevention do not support this prevention strategy because the evidence for it is weak [7]. Therefore, the appropriate therapy strategy for aspirin is still unclear.

There have been many large trials to detect the true effect of aspirin use; they all included diabetes patients without previous cardiovascular disease. A Study of Cardiovascular Events in Diabetes (ASCEND: 15,480 diabetic participants under 100 mg of aspirin once daily and placebo, median follow-up of 7.4 years), showed that individuals using aspirin had a 12% reduction in risk of major adverse cardiovascular events (MACE) but had a 29% increase in bleeding risk compared with a placebo group [8]. Another large trial, namely, the Aspirin in Reducing Events in the Elderly (ASPREE: 2057 diabetes patients with 100 mg aspirin once daily and placebo, median follow-up of 4.7 years) revealed a non-significant 10% reduction in the risk of MACE and a non-significant 30% increase in bleeding risk [9]. The other large study, namely the Japanese Primary Prevention of Atherosclerosis with Aspirin for Diabetes (JPAD2: 2160 diabetic participants with 81 or 100 mg daily and placebo, median follow-up of 10.3 years), which was a randomized, open-label, standard care-controlled trial, revealed no statistically significant benefit in cardiovascular events [10]. These large randomized control trials indicated that aspirin may have a positive effect on the prevention of CVD, but the increased bleeding risk should be considered. Furthermore, these results were compatible with results from the previous meta-analysis, which also showed inconsistent results about the role of aspirin in primary prevention of CVD and risk for bleeding among subjects with diabetes [11,12,13,14,15]. The possible explanation for this discrepancy may be attributable to several factors, such as dose of aspirin, age, gender, or ethnic factors.

The dosage of aspirin used in previous studies ranged from 100 mg every other day to 650 mg per day, which may have led to a selection bias and affected the results of the meta-analysis. Given the practical problem of aspirin use in diabetes, we aimed to focus on low-dose aspirin use in diabetes for primary prevention in this investigation. Owing to the recent publication of large randomized control trials, including JPAD2, ASCEND, and ASPREE trials, we aimed to effectively summarize the eligible evidence and promote the proper dissemination of this important clinical information. To accomplish this goal in our meta-analysis, we used the GRADE profiler guideline development tool software to assess the overall certainty of the evidence in this topic area. Statistical methods were used to evaluate the benefits and disadvantages of aspirin use in diabetes and to make an objective conclusion regarding its use as the primary prevention method for CVD in diabetic patients.

## 2. Experimental Section

### 2.1. Data Sources and Searches

We performed a systematic literature search in electronic datasets (i.e., PubMed, Embase, and Cochrane central) for randomized controlled trials (RCTs) that evaluated low-dose aspirin as the primary prevention strategy in diabetes (additional search details are listed in Table S2).

**Figure 1:**
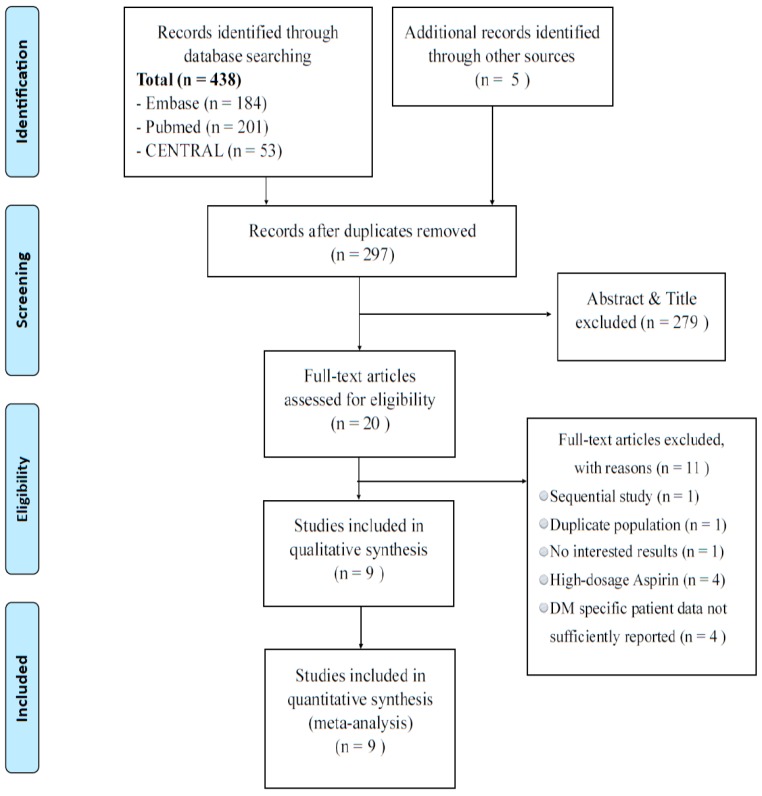
PRISMA flowchart of study selection. Flow diagram of the identification process for eligible studies.

We also conducted manual screening for references from original articles, conference abstracts, and previous systematic reviews to identify eligible trials. We followed the Preferred Reporting Items for Systematic Reviews and Meta Analyses (PRISMA) guidelines for performing the systematic reviews and meta-analyses of RCTs (Figure 1). The protocol for this systematic review is registered with PROSPERO (no. CRD4201811830).

### 2.2. Study Selection

Low-dose aspirin was defined as a daily aspirin regimen (≤100 mg). Patients with either type 1 or type 2 diabetes without previous major vascular events were included. Furthermore, peer-reviewed RCTs written in various languages were included. Trials examining aspirin use as the primary prevention strategy that had subgroups with diabetes were also included. Language with English or Chinese were included.

Trials identified from the literature search were initially screened for relevance based on titles and abstracts by two independent reviewers (Cho-Hao Lee and Chin Lin). Studies that did not meet these criteria during the title and abstract screen were excluded. Full-text reviews were then performed using the studies included after screening to ensure that they met the eligible criteria. Any disagreement between the two independent reviewers was resolved via group discussions.

### 2.3. Data Extraction and Quality Assessment

Two independent reviewers (Cho-Hao Lee and Chin Lin) extracted various data, including author names, publication year, geographic regions, trial names, study designs, sample sizes, and participant characteristics (mean age, sex, inclusion criteria, aspirin dosage, follow-up duration, and completion). The outcomes of interest including MACE (a composite of non-fatal myocardial infarction, non-fatal stroke, and death from cardiovascular causes), major hemorrhage (major intracranial hemorrhage and major gastrointestinal bleeding), and all-cause mortality were also extracted. To address the risk of bias with multiple data extractors, standardized “Google Excel” templates were created. Last, the third reviewer (Ming-Hsun Lin) double-checked the data for accuracy.

The quality of trials was appraised using the *Cochrane Handbook for Systematic Reviews of Interventions* [16]. Seven domains—selection bias, attrition bias, performance bias, detection bias, reporting bias, contamination bias, and other risks of bias—are listed in the Figure S1. Any disagreement between the two reviewers was resolved via group discussions.

### 2.4. Data Synthesis and Analysis

Data analysis was conducted as recommended in the *Cochrane Handbook for Systematic Reviews of Interventions* [17]. We used both random and fixed effects modeling to pool all outcomes and interpreted random-effects meta-analyses with consideration to the complete distribution of effects. We calculated dichotomous outcomes by using the Mantel–Haenszel method and evaluated the risk ratio (RR) with 95% CIs.

Heterogeneity and publication biases were evaluated by *I*^2^ statistic and funnel plots with Egger’s test. Statistically significant heterogeneity was defined as an *I*^2^ statistic > 50%. The cause of heterogeneity was investigated for main outcomes by using sensitivity tests and a mixed-effects meta-regression model with variables including follow-up duration, mean age, sample sizes, country, completion, and publication year [17]. All statistical analyses were performed using the ‘metafor’ and ‘meta’ [18] packages of R software version 3.3.1 [19]. A two-tailed significance test (*p* value = 0.05) denoted statistical significance without multiplicity correction in all exploratory analyses.

Furthermore, to assess the certainty of the evidence for each outcome, we used the GRADE profiler guideline development tool software [20] and ranked the quality of the evidence according to the *Cochrane Handbook for Systematic Reviews of Interventions* [16,21]. This method takes issues related to internal validity (e.g., risk of bias, inconsistency, imprecision, and publication bias) and external validity (e.g., directness of results) into account. We downgraded the evidence from “high” certainty by one level for serious concerns and by two levels for very serious concerns.

A trial sequential analysis (TSA), which is similar to interim analyses in a single trial, was also conducted [22,23]. Monitoring boundaries were used to decide if a trial could be terminated early (i.e., when a p value was sufficiently small to show the anticipated effect). We also used monitoring boundaries to determine if a study produced a sufficiently small p value to demonstrate the anticipated power. The chance of random errors may have been increased owing to insufficient comparisons and the repetitive testing of pooled data when the estimated information samples had not been achieved [22,24,25]. TSA version 0.9 beta [26] was used for the quantification of information samples.

## 3. Results

The systematic search yielded 443 studies, and 297 studies remained after removing duplicates. Then 279 studies were excluded after reviewing the abstract and title. There were 20 eligibility full-text articles; 11 of them were excluded for several reasons after reviewing the full-text articles. The remaining nine randomized control trials involving 29,814 participants were included (Figure 1). Among the total sample, 14,897 patients were randomized to low-dose aspirin use, and 14,917 patients were randomized to a control group. Table 1 shows the characteristics of each study. Four of the nine trials contained subgroups with diabetes, and the other five trials were studies that primarily focused on diabetes. These trials were published from 1998 to 2018 and were conducted in more than five different countries; the mean follow-up period ranged from 3.6 years to 10.3 years. The dosage of aspirin used ranged from 100 mg/every other day to 100 mg/daily.

Meta-regression analyses were performed for MACE, myocardial infarction (MI), stroke, major hemorrhage, and all-cause mortality (see Table S3). There was no significant association of aspirin use with the above outcomes. However, although it was not surprising, all-cause mortality and mean age were related.

### 3.1. Major Adverse Cardiovascular Event and Major Hemorrhage

**Figure 2:**
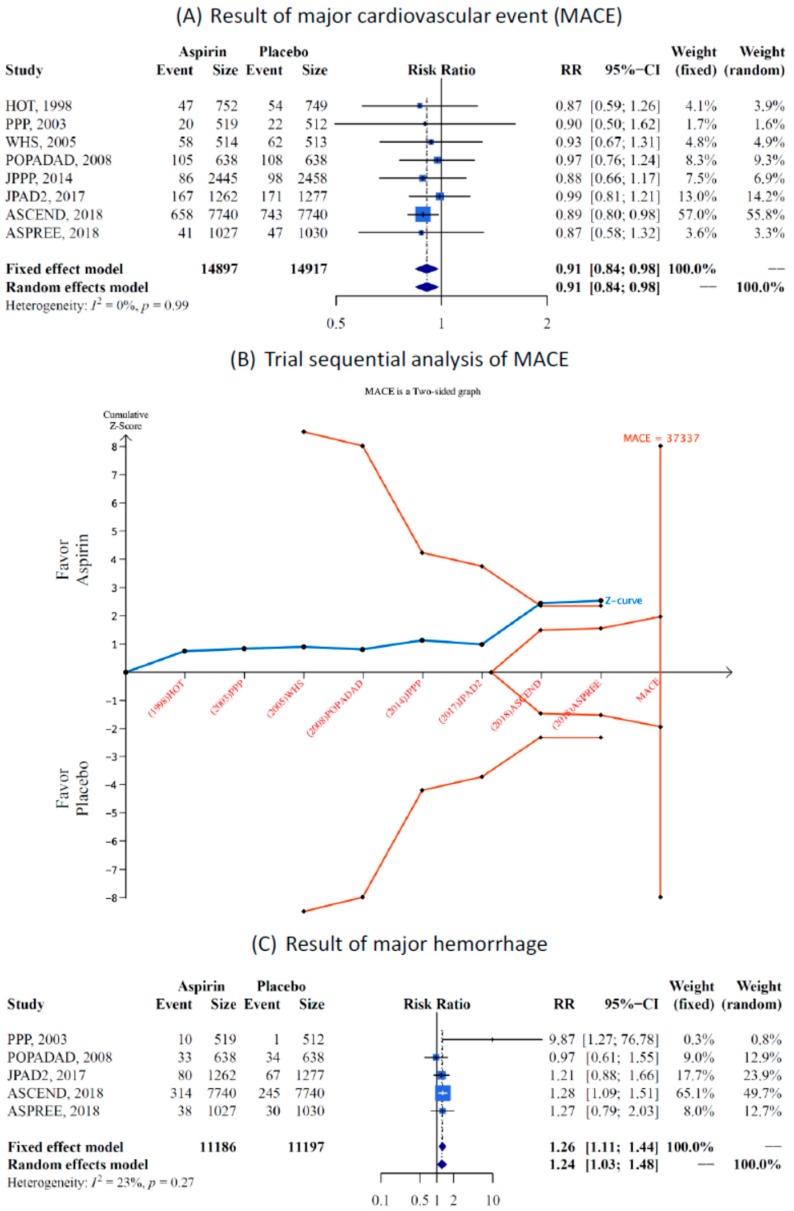
Meta-analysis result, (**A**) Forest plot of major adverse cardiovascular events (MACE) in aspirin compared with control, (**B**) Trial sequential analysis (TSA) of MACE, heterogeneity adjustment required an information size of 37,337 participants calculated on the basis of the proportion of MACE incidence of 8.7% in the placebo group, as well as α = 5%, β = 20%, power = 0.80, and *I*^2^ = 0.00%. Cumulative Z-curve (solid blue line) crosses the trial sequential monitoring boundary, which shows sufficient evidence of a statistically significant reduction in MACE risk with low-dose aspirin administration after involving large trials such as ASCEND [8] and ASPREE [9]. Horizontal dark red lines illustrate the traditional level of statistical significance (*p* = 0.05), (**C**) forest plot of major hemorrhage in aspirin compared with control. Main outcomes of low-dose aspirin in the primary prevention of cardiovascular disease (CVD). Risk ratio and 95% CI were used as a measure of effect for dichotomous variables. (Trial abbreviations are listed in the footnote of Table 1).

This study revealed a significant reduction in the risk of MACE (Figure 2A) in the low-dose aspirin group compared with the placebo and no-treatment groups (eight studies, RR 0.91, 95% CI 0.84–0.98). No heterogeneity was found between the studies (*I*^2^ = 0%, *p* value = 0.99). Furthermore, TSA was used to analyze MACE. After including the three large trials (JPAD2, ASCEND, and ASPREE), the analysis showed a significant reduction in the risk of MACE with low-dose aspirin administration (Figure 2B).

The GRADE system demonstrated a moderate level recommendation for low-dose aspirin administration in diabetic patients (see Table 2).

Major hemorrhaging, including intracranial bleeding and gastrointestinal (GI) bleeding, was the most important adverse effect of aspirin use. The results revealed a significant increase in hemorrhage risk (24%) with aspirin administration (five studies, RR = 1.24, 95% CI =1.03–1.48). The heterogeneity between studies in this analysis was low (*I*^2^ = 23%, *p* value = 0.27, see Figure 2C).

### 3.2. Efficacy and Safety End Point

We analyzed several secondary outcomes, including MI, coronary heart disease, and stroke, and summarized the results in Table 2.

There was no reduction in the risk of MI (six studies, RR = 1.01, 95% CI = 0.84–1.22, see Table 2 and Figure S2) or coronary heart disease (six studies, RR = 0.94, 95% CI = 0.84–1.06, see Table 2 and Figure S2). The heterogeneity of evidence was low for MI (*I*^2^ = 21%, *p* value = 0.27), and no heterogeneity of evidence was found for coronary heart disease (*I*^2^ = 0%, *p* value = 0.86).

Additionally, there was a reduction in the risk of stroke with aspirin use (seven studies, RR = 0.84, 95% CI = 0.73–0.97, see Table 2 and Figure S2). No heterogeneity of evidence was found in this analysis (*I*^2^ = 0%, *p* value = 0.65). No significant reduction in the risk of cardiovascular death (five studies, RR = 0.94, 95% CI = 0.77–1.14, see Table 2 and Figure S2) or all-cause mortality (six studies, RR = 0.97, 95% CI = 0.90–1.06, see Table 2 and Figure S2) was found. Furthermore, there was no heterogeneity of evidence in cardiovascular death (*I*^2^ = 0%, *p* value = 0.54) or all-cause mortality (*I*^2^ = 0%, *p* value = 0.46).

This present study showed increased hemorrhagic risk by 24% in the aspirin group. The subgroup analyses of major hemorrhage events revealed no statistically significant difference in aspirin use regarding major gastro-intestinal or intra-cranial hemorrhage (Table 2).

### 3.3. Sensitivity Tests and Publication Bias

We used sensitivity tests, subgroup analyses, and meta-regression analyses to explore potential heterogeneity. Meta-regression analyses were performed for MACE, myocardial infarction (MI), stroke, major hemorrhage, and all-cause mortality (see Figure S2). There was no significant association of aspirin use with the above outcomes. There was no sex difference in our present study. However, although it was not surprising, all-cause mortality and mean age were related. Publication bias was evaluated by visual examination and Egger’s regression test for all interested outcomes. Neither visual examinations nor Egger‘s regression test showed that significant publication bias existed (see Figure S3).

## 4. Discussion

This present study demonstrated that use of low dose aspirin in diabetic patients as primary prevention of cardiovascular disease is warranted. Our sophisticated analysis showed a moderate level recommendation to use aspirin for reducing the risk of MACE. Although aspirin use increased the risk of major hemorrhage (i.e., major GI bleeding and major intracranial bleeding), it could be prescribed among subjects of diabetes without obvious bleeding risk, or otherwise other research has suggested that the use of proton pump inhibitors (PPIs) can decrease the risk of GI bleeding from long-term aspirin use [34]. Long-term aspirin therapy achieves the benefit of MACE prevention with a number needed to treat per year of 122 and carries a risk of GI hemorrhage with a number needed to harm per year of 115. It is possible to estimate the balance between the benefit and harm for aspirin use in diabetic patients with different cardiovascular risk levels by comparing the pooled estimate of the number needed to harm against the number needed to treat from individual trials.

The results confirmed the current guideline of ADA that low dose aspirin may be considered as a primary prevention strategy in those with diabetes mellitus (DM) who are at increased cardiovascular risk and are not at increased risk of bleeding [6].

Age is an important factor to determine the usage of low dose aspirin in the primary prevention of CVD in subjects of DM. A subgroup analysis was performed for those aged 60 years and older. For this subgroup, aspirin use also showed a protective effect in major adverse cardiovascular events compared with the control group (RR = 0.91, CI = 0.83–0.98, Table 2). However, GRADE analysis showed that the recommendation level was low. For those younger than 60 years old, there was no statistically significant effect in MACE (RR = 1.00, CI = 0.73–1.93, Table 2). This could be attributable to the high heterogeneity between studies (*I*^2^ = 67.2%) and the low power. In conclusion, the benefit only existed among subjects aged greater than 60 years old instead of below 60 years old in our subgroup analysis. Elderly diabetic populations may have more cardiovascular risk factors, which may explain this difference.

### 4.1. Comparison of Previous Published Meta-Analysis

This study only included low-dosage aspirin (less than 100 mg daily) and used trial sequential analysis to assess the true results compared with previous published studies. Our finding of a 9% reduction in the risk of MACE is similar to previously published meta-analyses published between 2009 and 2016 with a non-significant 8 to 10% reduction in MACE [5,14,15,35,36]. The present meta-analysis enrolled a large sample size and was adequate to detect the benefit of aspirin in the prevention of CVD among subjects of diabetes. Furthermore, the present study included primary and secondary end points that revealed little to no heterogeneity of evidence, further supporting the important role of aspirin.

Compared with previous work, this analysis showed no statistically significance reduction of MI in the aspirin group, which was similar to previous published meta-analysis that revealed a non-statistically significance 14% to 17% reduction in MI. Although previous studies revealed the trend of decreased risk, our study showed a neutral effect after including a large trial of ASCEND, which enrolled the most diabetic population. In contrast, the individual participant meta-analysis reported that aspirin may significantly reduce the incidence of MI [2]. Further research is warranted.

Previous published meta-analyses reported a trend for the reduction in stroke risk, and the mean RR reduction ranged from 14% to 30% [4,5,11,12,13,14,35]. Our analysis demonstrated a moderate level of evidence for a reduction in stroke risk with low-dose aspirin administration. The adequacy of the present investigation may explain the difference between studies. For major bleeding risk, all previous results showed increased bleeding risk ranging from 54% to 69%, but no statistical significance. Our present study also showed similar results that statistically significantly increased bleeding risk by 24%. Furthermore, the bleeding risk in our study was lower than previous results because of three large trials included (JPAD2, ASCEND, and ASPREE).

### 4.2. Strengths and Limitations

The major strength of the present analysis is the focus on the low dose of aspirin in the primary prevention of CVD among subjects with diabetes. Besides, owing to the well-designed protocol and detailed inclusion criteria, we conducted the largest meta-analysis of low-dose aspirin in diabetic patients to date (nearly 30,000 people). This large sample allowed the examination of primary points, secondary points, efficacy, and safety. To further clarify the effect of aspirin, we also performed evaluations of internal validity (risk of bias, inconsistency, imprecision, and publication bias) and external validity (directness of results). Even though the risk of hemorrhage increased, our analysis is the first analysis to show a significant reduction of MACE with aspirin use for diabetic patients.

One limitation of the current investigation is the exclusion of observational studies, which may provide more real-world data and longer follow-up times. Compared with RCT, the outcomes of real-world evidence (RWE) continue to be assigned lower credibility. It must be emphasized that RWE research is a real-world practice that does not need to be executed as RCT research for it to be reliable. RCT research, characterized as having the highest reliability, and RWE research, which reflects the actual clinical aspects, can have a mutually supplementary relationship. We believe that our present meta-analysis of randomized control trial data proved the efficacy and safety of low dose aspirin; however, we still need large real-world evidence to inspect the effect on clinical practice [37].

Furthermore, the diversity of the sample is low because five of the trials were conducted in the United States, two were in Europe (United Kingdom and Italy), three were in Japan, and only one study utilized a global design. Therefore, the generalizability of these results to other countries, races, and ethnicities is limited.

An unpublished large trial still in progress, namely the Aspirin and Simvastatin Combination for Cardiovascular Events Prevention Trial in Diabetes (ISRCTN48110081), enrolled approximately 50,000 participants and followed participants for approximately 5 years [38]. This trial will help clarify the benefits of aspirin use. Although previous randomized control trials exist, they primarily include Caucasian and Japanese participants. Additional large-scale trials with a global design are necessary to better understand the benefits and disadvantages of aspirin use in diabetic individuals around the world.

## 5. Conclusions

Previous published data do not recommend aspirin use as the primary prevention strategy for diabetic individuals. The current investigation has shown a statistically significant protective effect and a moderate level of confidence for the administration of low-dose aspirin for the primary prevention in diabetes, particularly in older individuals (age ≥ 60 years). However, clinicians should assess the effect of aspirin use in each patient on a case-by-case basis and use evidence-based medicine to guide clinical decision making, especially considered the absolute benefits were largely counterbalanced by the bleeding hazard.

## Figures and Tables

**Table 1:** Characteristics of the included randomized control trials.

Source	Trial Design	Country (Study Name)	Population (DM)	Case (Mean Age)	Males	Aspirin Dosage	Mean Follow up (Completion Rate)
MRC 1998 [27]	PC, RCT	UK (TPT)	Patients at high risk of IHD (type 1 and 2)	68 (57.3 years *)	100.0	75 mg/daily	6.7 years/98.9
Hansson et al. 1998 [28]	DB, RCT	Globally (HOT)	Participants with hypertension (type 1 and 2)	1501 (61.5 years)	NR	75 mg/daily	3.8 years/97.4
Sacco et al. 2003 [29]	MC, OP, RCT, 2 × 2 factorial design	Italy (PPP)	Participants > 50 years with > one cardiovascular risk factor (type 2)	1031 (64.2 years)	48.2	100 mg/daily	3.6 years/99.3
Ridker et al. 2005 [30]	MC, DB, RCT, 2 × 2 factorial design	USA (WHS)	Healthy women (NA)	1027 (54.6 years *)	0	100 mg/every other day	10.1 years/99.4
Belch et al. 2008 [31]	DB, RCT, 2 × 2 factorial design	UK (POPADAD)	Patients aged ≥ 40 years plus ABP ≤ 0.99 (type 1 and 2)	1276 (60 years)	44.1	100 mg/daily	6.7 years/99.5
Ikeda et al. 2014 [32]	OP, RCT	Japan (JPPP)	Elderly with multiple atherosclerotic risk factors (NR)	4903 (70.6 years *)	NR	100 mg/daily	5.0 years/98.7
Saito et al. 2017 [10]	MC, OP, RCT	Japan (JPAD2)	Patients with Type 2 Diabetes (type 2)	2160 (64.4 years)	55.5	81 or 100 mg/daily	10.3 years/63.8
ASCEND group 2018 [8]	DB, RCT	UK (ASCEND)	Patients with diabetes (type 1 and 2)	15,800 (63.3 years)	62.6	100 mg/daily	7.4 years/99.1
McNeil et al. 2018 [9]	DB, RCT	USA/Australia (ASPREE)	Health older (NR)	2057 (74 years *)	44	100 mg/daily	4.7 years/97.2

DM: diabetes mellitus; MC: multi-center; DB: double blind; OP: open label; RCT: randomized control trial; NR: Not reported; ABP: Ankle brachial pressure; TPT: thrombosis prevention trial; HOT: hypertension optimal treatment; PPP: primary prevention project; WHS: women’s health study; WHS: women’s health study; POPADAD: prevention of progression of arterial disease and diabetes; JPAD2: Japanese primary prevention of atherosclerosis with aspirin for diabetes 2, followed up study from JPAD [33]; JPPP: Japanese primary prevention project; ASCEND: a study of cardiovascular events in diabetes; ASPREE: Aspirin in reducing events in the elderly. *: data extracted from specific diabetes patients.

**Table 2:** Effects of low-dose aspirin use as the primary prevention strategy on clinical outcomes in diabetic patients.

Outcome Assessment	No. of Trials (Patients)	Anticipated Absolute Effects (95% CI)		Risk Ratio (95% CI) Random-Effect Estimate	*p*-Value Random-Effect Estimate	Heterogeneity *I*^2^(%) Cochrane Q *p*-Value	Certainty of Evidence (GRADE) ^†^
		Risk with Placebo	Risk with Aspirin				
MACE	8 (29,814)	87 per 1000	80 per 1000 (73 to 86)	**0.91 (0.84–0.98)**	0.018 *	0.0% *p* = 1.00	⨁⨁⨁◯ MODERATE
MACE with age ≥ 60 years	5 (18,664)	111 per 1000	101 per 1000 (92 to 108)	**0.91 (0.83–0.98)**	0.023 *	0.0% *p* = 1.00	⨁⨁◯◯ LOW
MACE with age < 60 years	3 (7212)	90 per 1000	91 per 1000 (65 to 124)	1.00 (0.73–1.39)	0.973	67.2% *p* = 0.0476	⨁◯◯◯ Very LOW
Myocardial infarction	6 (22,854)	31 per 1000	32 per 1000 (26 to 38)	1.01 (0.84–1.22)	0.891	21.1% *p* = 0.275	⨁⨁◯◯ LOW
Fatal myocardial infarction	3 (19,295)	15 per 1000	14 per 1000 (9 to 23)	0.98 (0.60–1.60)	0.942	44.1% *p* = 0.166	⨁◯◯◯ Very LOW
Stroke	7 (22,922)	36 per 1000	31 per 1000 (27 to 35)	**0.84 (0.73–0.97)**	0.017 *	0.0% *p* = 0.645	⨁⨁⨁◯ MODERATE
Fatal stroke	3 (19,025)	5 per 1000	6 per 1000 (4 to 9)	1.20 (0.82–1.77)	0.349	0.0% *p* = 0.773	⨁◯◯◯ Very LOW
Coronary heart disease	6 (22,923)	48 per 1000	45 per 1000 (40 to 50)	0.94 (0.84–0.1.06)	0.323	0.0% *p* = 0.864	⨁⨁◯◯ LOW
Major hemorrhage	5 (22,383)	34 per 1000	42 per 1000 (35 to 50)	**1.24 (1.03–1.48)**	0.0221 *	22.9% *p* = 0.2687	⨁⨁◯◯ LOW
Major intracranial hemorrhage	5 (21,353)	6 per 1000	7 per 1000 (5 to 9)	1.08 (0.77–1.50)	0.654	0.0% *p* = 0.666	⨁⨁◯◯ LOW
Major Gl bleeding	4 (20,326)	14 per 1000	20 per 1000 (13 to 32)	1.43 (0.92–2.22)	0.117	56.7% *p* = 0.074	⨁⨁◯◯ LOW
All-cause death	6 (23,884)	87 per 1000	84 per 1000 (78 to 92)	0.97 (0.90–1.06)	0.537	0.0% *p* = 0.457	⨁⨁◯◯ LOW
Cardiovascular death	5 (21,827)	18 per 1000	17 per 1000 (14 to 21)	0.94 (0.77–1.14)	0.517	0.0% *p* = 0.545	⨁⨁◯◯ LOW

MACE: major adverse cardiovascular event; GI: gastrointestinal; CI: confidence interval; *I*^2^: index for assessing heterogeneity; value > 50% indicates a moderate to high heterogeneity. *: The significance level in the classical model was set as <0.05; ^†^: Certainty of effect estimates: ⊕⊕⊕⊕, high; ⊕⊕⊕, moderate; ⊕⊕, low; ⊕, very low.

## Supplementary Materials

The following are available online at https://www.mdpi.com/2077-0383/8/5/609/s1, Table S1. PRISMA checklist, Table S2. Search details, Table S3. Meta-regression results, Figure S1. Assessment of risk of bias, Figure S2. Forest plots of secondary outcomes, Figure S3. Funnel plots and the results of Egger’s test of major outcomes in participants with diabetes for aspirin intervention trials.
