# Supplementary material for: Low-Dose Aspirin for the Primary Prevention of Cardiovascular Disease in Diabetic Individuals: A Meta-Analysis of Randomized Control Trials and Trial Sequential Analysis
Source: J Clin Med. 2019 May 5;8(5):609. doi: 10.3390/jcm8050609 (PMC6572181; doi:10.3390/jcm8050609)
Supplement: Supplementary file 1 [file jcm-08-00609-s001.pdf]

Journal of
*Clinical Medicine*

MDPI

*Article*

# Low-Dose Aspirin for the Primary Prevention of Cardiovascular Disease in Diabetic Individuals: A Meta-Analysis of Randomized Control Trials and Trial Sequential Analysis

**Ming-Hsun Lin [1], Chien-Hsing Lee [1], Chin Lin [2,3], Yi-Fen Zou [4], Chieh-Hua Lu [1,5], Chang-Hsun Hsieh [1,*] and Cho-Hao Lee [6,*]**

## Supplementary materials

Table S1. PRISMA checklist.
Table S2. Search details.
Table S3. Meta-regression results.
Reference List of included full-text screening studies.
Figure S1. Assessment of risk of bias.
Figure S2. Forest plots of secondary outcomes.
Figure S3. Funnel plots and the results of Egger's test of major outcomes in participants with diabetes for aspirin intervention trials.

**Table 1.** PRIMSA checklist.

| Section/topic | Item No. | Checklist item | Reported on page No. |
|---|---|---|---|
| | | **Title** | |
| Title | 1 | Identify the report as a systematic review, meta-analysis, or both | 1 |
| | | **Abstract** | |
| Structured summary | 2 | Provide a structured summary including, as applicable, background, objectives, data sources, study eligibility criteria, participants, interventions, study appraisal and synthesis methods, results, limitations, conclusions and implications of key findings, systematic review registration number | 4-5 |
| | | **Introduction** | |
| Rationale | 3 | Describe the rationale for the review in the context of what is already known | 6-8 |
| Objectives | 4 | Provide an explicit statement of questions being addressed with reference to participants, interventions, comparisons, outcomes, and study design (PICOS) | 6-8 |
| | | **Methods** | |
| Protocol and registration | 5 | Indicate if a review protocol exists, if and where it can be accessed (such as web address), and, if available, provide registration information including registration number | 9 |
| Eligibility criteria | 6 | Specify study characteristics (such as PICOS, length of follow-up) and report characteristics (such as years considered, language, publication status) used as criteria for eligibility, giving rationale | 9 |
| Information sources | 7 | Describe all information sources (such as databases with dates of coverage, contact with study authors to identify additional studies) in the search and date last searched | 9 |
| Search | 8 | Present full electronic search strategy for at least one database, including any limits used, such that it could be repeated | Appendices |
| Study selection | 9 | State the process for selecting studies (that is, screening, eligibility, included in systematic review, and, if applicable, included in the meta-analysis) | 9 |

| | | | |
|---|---|---|---|
| Data collection process | 10 | Describe method of data extraction from reports (such as piloted forms, independently, in duplicate) and any processes for obtaining and confirming data from investigators | 10 |
| Data items | 11 | List and define all variables for which data were sought (such as PICOS, funding sources) and any assumptions and simplifications made | 10 |
| Risk of bias in individual studies | 12 | Describe methods used for assessing risk of bias of individual studies (including specification of whether this was done at the study or outcome level), and how this information is to be used in any data synthesis | 11 |
| Summary measures | 13 | State the principal summary measures (such as risk ratio, difference in means). | 10-11 |
| Synthesis of results | 14 | Describe the methods of handling data and combining results of studies, if done, including measures of consistency (such as $I^2$ statistic) for each meta-analysis | 10-11 |
| Risk of bias across studies | 15 | Specify any assessment of risk of bias that may affect the cumulative evidence (such as publication bias, selective reporting within studies) | 10-11 |
| Additional analyses | 16 | Describe methods of additional analyses (such as sensitivity or subgroup analyses, meta-regression), if done, indicating which were pre-specified | 10-11 |
| | | **Results** | |
| Study selection | 17 | Give numbers of studies screened, assessed for eligibility, and included in the review, with reasons for exclusions at each stage, ideally with a flow diagram | 12, Figure 1 |
| Study characteristics | 18 | For each study, present characteristics for which data were extracted (such as study size, PICOS, follow-up period) and provide the citations | 12, Table 1 |
| Risk of bias within studies | 19 | Present data on risk of bias of each study and, if available, any outcome-level assessment (see item 12). | 12, Appendices |
| Results of individual studies | 20 | For all outcomes considered (benefits or harms), present for each study (a) simple summary data for each intervention group and (b) effect estimates and confidence intervals, ideally with a forest plot | 12-14, Table 2 |
| Synthesis of results | 21 | Present results of each meta-analysis done, including confidence intervals and measures of consistency | 10-12, Figures 1-2; Table 2 |
| Risk of bias across studies | 22 | Present results of any assessment of risk of bias across studies (see item 15) | 10-12, Figures 1-2; Table 2 |
| Additional analysis | 23 | Give results of additional analyses, if done (such as sensitivity or subgroup analyses, meta-regression) (see item 16) | Appendices |
| | | **Discussion** | |
| Summary of evidence | 24 | Summarise the main findings including the strength of evidence for each main outcome; consider their relevance to key groups (such as health care providers, users, and policy makers) | 13-15 |
| Limitations | 25 | Discuss limitations at study and outcome level (such as risk of bias), and at review level (such as incomplete retrieval of identified research, reporting bias) | 15 |
| Conclusions | 26 | Provide a general interpretation of the results in the context of other evidence, and implications for future research | 16 |
| | | **Funding** | |
| Funding | 27 | Describe sources of funding for the systematic review and other support (such as supply of data) and role of funders for the systematic review | None |

**Table 2.** Search details.

| Ovid MEDLINE | EMBASE (OVID) | The Cochrane Library |
|---|---|---|
| 1.exp Primary Prevention/<br>2.primary prevention.ab,ti.<br>3.1 or 2<br>4.exp Diabetes mellitus/<br>5.diabetes mellitus.ab,ti.<br>6.4 or 5<br>7.exp Aspirin/<br>8.aspirin.ab,ti.<br>9.7 or 8<br>10.3 and 9<br>11.6 and 9<br>12.10 or 11<br>13.(controlled clinical trial or randomized controlled trial or meta analysis).pt.<br>14.(placebo* or random* or trial* or groups).ti,ab.<br>15.drug therapy.fs.<br>16.13 or 14 or 15<br>17.limit 16 to animals<br>18.limit 16 to (animals and humans)<br>19.17 not 18<br>20.16 not 19<br>21.12 and 20 | 1. exp Primary Prevention/<br>2. primary prevention.ab,ti.<br>3. 1 or 2<br>4. exp Diabetes mellitus/<br>5. diabetes mellitus.ab,ti<br>6. 4 or 5<br>7. exp Aspirin/<br>8. aspirin.ab,ti.<br>9. 7 or 8<br>10. 3 and 9<br>11. 6 and 9<br>12. 10 or 11<br>13. cross-over procedure/ or double-blind procedure/ or randomized controlled trial/ or single-blind procedure/<br>14. (allocat* or assign* or cross over* or crossover* or (double ADJ blind*) or factorial or placebo* or random* or (single ADJ blind*) or volunteer*). ti,ab.<br>15. 13 or 14<br>16. limit 15 to animals<br>17. limit 15 to (animals and humans)<br>18. 16 not 17<br>19. 15 not 18<br>20. 12 and 19 | 1.(aspirin): ti,ab,kw<br>2.(primary prevention): ti,ab,kw<br>3.(diabetes mellitus): ti,ab,kw<br>4.(#1 AND #2)<br>5.(#1 AND #3)<br>6.(#4 OR #5)<br>7.Restricted to "Cochrane Reviews", "Other Reviews", and "Clinical Trials" |

Relevant studies, published from inception to November 10, 2018 (date last searched), were identified through electronic searches not limited to the English language using Medline, EMBASE, Web of Science, and Cochrane databases. Electronic searches were supplemented by scanning reference lists of articles identified for all relevant studies (including review articles), and by hand searching of relevant journals. The computer-based searches combined search terms related to (1) the intervention (low dose aspirin, *salicylic acid and salicylates*) and (2) diabetes (*diabetes mellitus, type 2 diabetes, and type 1 diabetes*) or primary prevention.

**Table 3.** meta-regression results of main outcomes.

| Moderators | Variables | Study Number (N) | $RR_{interaction}$ (95% CI) | P-value | Cochrane Q/df | $I^2$ (%) |
|---|---|---|---|---|---|---|
| **MACE** | Follow up duration | 8 | 1.017 (0.974 to 1.062) | 0.4521 | 0.8277 | 0.00% |
| | Mean Age | 8 | 0.996 (0.976 to 1.018) | 0.7412 | 1.2839 | 0.00% |
| | Numbers | 8 | 1.000 (1.000 to 1.000) | 0.9329 | 1.3858 | 0.00% |
| | Country | 8 | 0.942 (0.785 to 1.132) | 0.5250 | 0.9888 | 0.00% |
| | Compliance | 8 | 0.993 (0.978 to 1.009) | 0.3953 | 0.6705 | 0.00% |
| | Sex difference | 8 | 0.972 (0.688 to 1.372) | 0.8704 | 1.3663 | 0.00% |
| | Sex dominate | 8 | 1.031 (0.863 to 1.233) | 0.7349 | 1.2783 | 0.00% |
| | Publication Year | 8 | 0.998 (0.985 to 1.012) | 0.8285 | 1.3460 | 0.00% |
| **Myocardia Infarction** | Follow up duration | 6 | 1.089 (0.987 to 1.202) | 0.0899 | 3.4550 | 0.00% |
| | Mean Age | 6 | 0.954 (0.903 to 1.007) | 0.0888 | 3.4337 | 0.00% |
| | Numbers | 6 | 1.000 (0.999 to 1.000) | 0.5181 | 5.5841 | 28.37% |
| | Country | 6 | 1.036 (0.550 to 1.954) | 0.9124 | 6.3093 | 36.60% |
| | Compliance | 6 | 1.006 (0.962 to 1.052) | 0.7896 | 6.2314 | 35.81% |
| | Sex difference | 6 | 0.654 (0.388 to 1.104) | 0.1120 | 3.8055 | 0.00% |
| | Sex dominate | 6 | 1.217 (0.818 to 1.809) | 0.3327 | 5.0509 | 20.81% |
| | Publication Year | 6 | 1.006 (0.972 to 1.041) | 0.7419 | 6.3190 | 36.70% |
| **stroke** | Follow up duration | 6 | 0.995 (0.923 to 1.073) | 0.8944 | 1.8715 | 0.00% |
| | Mean Age | 6 | 0.998 (0.967 to 1.030) | 0.9032 | 1.8743 | 0.00% |
| | Numbers | 6 | 1.000 (1.000 to 1.000) | 0.7100 | 1.7508 | 0.00% |
| | Country | 6 | 1.040 (0.730 to 1.482) | 0.8273 | 1.8415 | 0.00% |
| | Compliance | 6 | 1.004 (0.979 to 1.029) | 0.7793 | 1.8106 | 0.00% |
| | Sex difference | 6 | 1.052 (0.324 to 3.411) | 0.9327 | 1.8820 | 0.00% |
| | Sex dominate | 6 | 1.203 (0.885 to 1.635) | 0.2377 | 0.4949 | 0.00% |
| | Publication Year | 6 | 0.997 (0.976 to 1.019) | 0.7989 | 1.8242 | 0.00% |
| **Major hemorrhage** | Follow up duration | 5 | 0.974 (0.865 to 1.097) | 0.6652 | 5.0035 | 40.04% |
| | Mean Age | 5 | 1.011 (0.960 to 1.064) | 0.6898 | 5.0074 | 40.09% |
| | Numbers | 5 | 1.000 (0.999 to 1.001) | 0.9311 | 5.1576 | 41.83% |
| | Country | 5 | 1.031 (0.589 to 1.805) | 0.9156 | 5.1096 | 41.29% |
| | Compliance | 5 | 1.002 (0.962 to 1.043) | 0.9348 | 5.1199 | 41.40% |
| | Sex difference | 5 | NA (NA to NA) | NA | NA | NA |
| | Sex dominate | 5 | 0.896 (0.505 to 1.589) | 0.7074 | 4.7709 | 37.12% |
| | Publication Year | 5 | 1.002 (0.946 to 1.062) | 0.9337 | 4.9861 | 39.83% |
| **All-caused death** | Follow up duration | 3 | 0.857 (0.699 to 1.050) | 0.1365 | 1.2402 | 19.37% |
| | Mean Age | 3 | 1.043 (1.001 to 1.086) | 0.0458 | 0.0485 | 0.00% |
| | Numbers | 3 | 1.001 (1.000 to 1.002) | 0.0460 | 0.0554 | 0.00% |
| | Country | 3 | NA ( NA to NA) | NA | NA | NA |
| | Compliance | 3 | 0.789 (0.623 to 0.998) | **0.0483** | 0.1394 | 0.00% |
| | Sex difference | 3 | NA (NA to NA) | NA | NA | NA |
| | Sex dominate | 3 | 0.699 (0.308 to 1.588) | 0.3924 | 3.2483 | 69.22% |
| | Publication Year | 3 | 1.036 (0.955 to 1.125) | 0.3924 | 3.2483 | 69.22% |

Country : East or West

Sex difference: men, women or mixed

Sex dominate: men dominate or women dominate

$RR_{interaction}$: interaction effect calculated by meta-regression; positive direction indicates that possible moderators might strengthen the treatment success rate in Micafungin relative to extensive Azole medication.

[a]:The significant level was set as 0.05.

**Supplementary Materials: Reference list of full-text screening studies. Included studies (No.1 to No,9)**


1. Thrombosis prevention trial: randomised trial of low-intensity oral anticoagulation with warfarin and low-dose aspirin in the primary prevention of ischaemic heart disease in men at increased risk. The Medical Research Council's General Practice Research Framework. *Lancet* 1998; **351**(9098): 233-41.
2. Hansson L, Zanchetti A, Carruthers SG, et al. Effects of intensive blood-pressure lowering and low-dose aspirin in patients with hypertension: principal results of the Hypertension Optimal Treatment (HOT) randomised trial. HOT Study Group. *Lancet* 1998; **351**(9118): 1755-62.
3. Sacco M, Pellegrini F, Roncaglioni MC, et al. Primary prevention of cardiovascular events with low-dose aspirin and vitamin E in type 2 diabetic patients: results of the Primary Prevention Project (PPP) trial. *Diabetes Care* 2003; **26**(12): 3264-72.
4. Ridker PM, Cook NR, Lee IM, et al. A randomized trial of low-dose aspirin in the primary prevention of cardiovascular disease in women. *N Engl J Med* 2005; **352**(13): 1293-304.
5. Belch J, MacCuish A, Campbell I, et al. The prevention of progression of arterial disease and diabetes (POPADAD) trial: factorial randomised placebo controlled trial of aspirin and antioxidants in patients with diabetes and asymptomatic peripheral arterial disease. *BMJ* 2008; **337**: a1840..
6. Ikeda Y, Shimada K, Teramoto T, et al. Low-dose aspirin for primary prevention of cardiovascular events in Japanese patients 60 years or older with atherosclerotic risk factors: a randomized clinical trial. *JAMA* 2014; **312**(23): 2510-20.
7. Yoshihiko Saito, MD, PhD Sadanori Okada, MD, PhD Hisao Ogawa, MD, PhD Hirofumi Soejima, MD, PhD. Low-Dose Aspirin for Primary Prevention of Cardiovascular Events in Patients With Type 2 Diabetes Mellitus 10-Year Follow-Up of a Randomized Controlled Trial. Circulation. 2017;135:659–670. DOI: 10.1161/CIRCULATIONAHA.116.025760
8. Louise Bowman, M.D., Marion Mafham, M.D., Karl Wallendszus, M.Sc., Will Stevens, Ph.D., Georgina Buck, M.Sc. Effects of Aspirin for Primary Prevention in Persons with Diabetes Mellitus. N Engl J Med 2018;379:1529-39. DOI: 10.1056/NEJMoa1804988
9. J.J. McNeil, R. Wolfe, R.L. Woods, A.M. Tonkin, G.A. Donnan, M.R. Nelson. Effect of Aspirin on Cardiovascular Events and Bleeding in the Healthy Elderly. N Engl J Med 2018;379:1509-18. DOI: 10.1056/NEJMoa1805819
10. Ogawa H, Nakayama M, Morimoto T, et al. Low-dose aspirin for primary prevention of atherosclerotic events in patients with type 2 diabetes: a randomized controlled trial. *JAMA* 2008; **300**(18): 2134-41
11. J.J. McNeil, M.R. Nelson, R.L. Woods, J.E. Lockery, R. Wolfe, C.M. Reid. Effect of Aspirin on All-Cause Mortality in the Healthy Elderly. N Engl J Med 2018;379:1519-28. DOI: 10.1056/NEJMoa1803955
12. Peto R, Gray R, Collins R, et al. Randomised trial of prophylactic daily aspirin in British male doctors. *Br Med J (Clin Res Ed)* 1988; **296**(6618): 313-6.
13. Final report on the aspirin component of the ongoing Physicians' Health Study. Steering Committee of the Physicians' Health Study Research Group. *N Engl J Med* 1989; **321**(3): 129-35.
14. Aspirin effects on mortality and morbidity in patients with diabetes mellitus. Early Treatment Diabetic Retinopathy Study report 14. ETDRS Investigators. *JAMA* 1992; **268**(10): 1292-300.
15. Gaziano JM, Brotons C, Coppolecchia R, et al., on behalf of the ARRIVE Executive Committee. Use of aspirin to reduce risk of initial vascular events in patients at moderate risk of cardiovascular disease (ARRIVE): a randomised, double-blind, placebo-controlled trial. *Lancet*2018;Aug 26:[Epub ahead of print].
16. Fowkes FG, Price JF, Stewart MC, Butcher I, Leng GC, Pell AC, Sandercock PA, Fox KA, Lowe GD, Murray GD; Aspirin for Asymptomatic Atherosclerosis Trialists. Aspirin for prevention of cardiovascular events in a general population screened for a low ankle brachial index: a randomized controlled trial. JAMA. 2010;303:841–848. doi: 10.1001/jama.2010.221
17. Erkan D, Harrison MJ, Levy R, Peterson M, Petri M, Sammaritano L, Unalp-Arida A, Vilela V, Yazici Y, Lockshin MD. Aspirin for primary thrombosis prevention in the antiphospholipid syndrome: a randomized, double-blind, placebo-controlled trial in asymptomatic antiphospholipid antibody-positive individuals. Arthritis Rheum. 2007;56:2382–2391. doi: 10.1002/art.22663.
18. Catalano M, Born G, Peto R. Prevention of serious vascular events by aspirin amongst patients with peripheral arterial disease: randomized, double-blind trial. J Intern Med. 2007;261:276–284.

19. Landolfi R, Marchioli R, Kutti J, Gisslinger H, Tognoni G, Patrono C, Barbui T; European Collaboration on Low-Dose Aspirin in Polycythemia Vera Investigators. Efficacy and safety of low-dose aspirin in polycythemia vera. N Engl J Med. 2004;350:114–124. doi: 10.1056/NEJMoa035572
20. Côté R, Battista RN, Abrahamowicz M, Langlois Y, Bourque F, Mackey A. Lack of effect of aspirin in asymptomatic patients with carotid bruits and substantial carotid narrowing: The Asymptomatic Cervical Bruit Study Group. Ann Intern Med. 1995;123:649–655

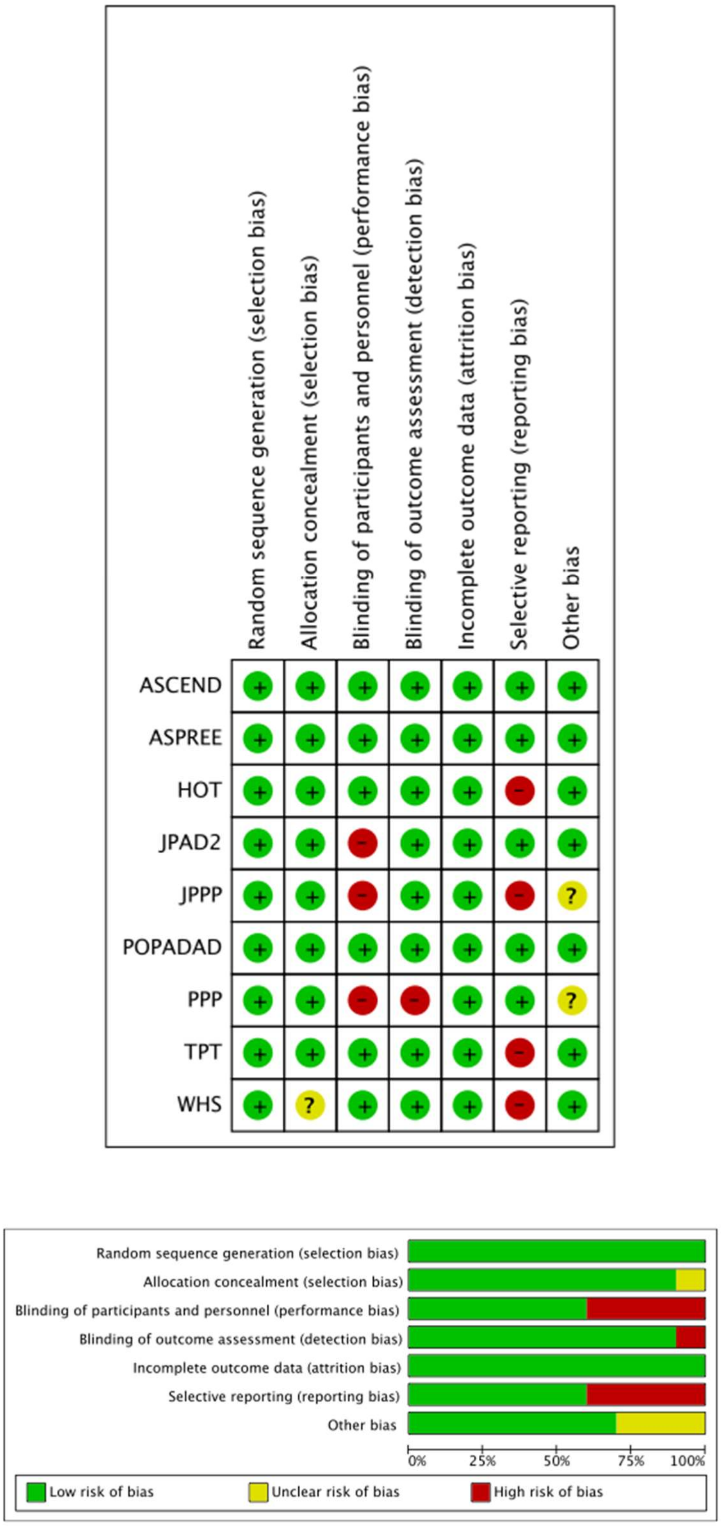


**Figure 1. Assessment of risk of bias.** TPT: Thrombosis prevention trial; HOT: Hypertension Optimal Treatment; PPP: Primary Prevention Project; WHS, Women's Health Study; WHS: Women's Health Study; POPADAD: Prevention of Progression of Arterial Disease and Diabetes; JPAD2: Japanese Primary Prevention of Atherosclerosis with Aspirin for Diabetes 2; JPPP: Japanese Primary Prevention Project; NR, not reported; JPAD: Japanese Primary Prevention of Atherosclerosis with Aspirin for Diabetes; ASCEND: A Study of Cardiovascular Events in Diabetes; ASPREE: Aspirin in Reducing Events in the Elderly.

Myocardial infarction

| Study | Aspirin Event | Aspirin Size | Placebo Event | Placebo Size | RR | 95%−CI | Weight (fixed) | Weight (random) |
|---|---|---|---|---|---|---|---|---|
| HOT, 1998 | 11 | 752 | 18 | 749 | 0.61 | [0.29; 1.28] | 5.0% | 5.6% |
| PPP, 2003 | 5 | 519 | 10 | 512 | 0.49 | [0.17; 1.43] | 2.8% | 2.8% |
| WHS, 2005 | 36 | 514 | 24 | 513 | 1.50 | [0.91; 2.47] | 6.7% | 11.4% |
| POPADAD, 2008 | 90 | 638 | 82 | 638 | 1.10 | [0.83; 1.45] | 22.9% | 27.9% |
| JPAD2, 2017 | 28 | 1262 | 29 | 1277 | 0.98 | [0.58; 1.63] | 8.1% | 10.9% |
| ASCEND, 2018 | 191 | 7740 | 195 | 7740 | 0.98 | [0.80; 1.19] | 54.5% | 41.3% |
| **Fixed effect model** | | **11425** | | **11429** | **1.01** | **[0.87; 1.16]** | **100.0%** | **--** |
| **Random effects model** | | | | | **1.01** | **[0.84; 1.22]** | **--** | **100.0%** |

Heterogeneity: $I^2 = 21\%$, $p = 0.27$

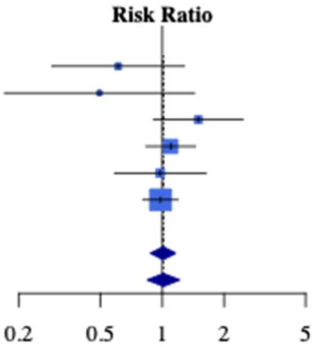


**Stroke**

| Study | Aspirin Event | Aspirin Size | Placebo Event | Placebo Size | RR | 95%−CI | Weight (fixed) | Weight (random) |
|---|---|---|---|---|---|---|---|---|
| TPT, 1998 | 1 | 29 | 2 | 39 | 0.67 | [0.06; 7.06] | 0.4% | 0.4% |
| HOT, 1998 | 20 | 752 | 22 | 749 | 0.91 | [0.50; 1.64] | 5.3% | 5.5% |
| PPP, 2003 | 9 | 519 | 10 | 512 | 0.89 | [0.36; 2.17] | 2.4% | 2.5% |
| WHS, 2005 | 15 | 514 | 31 | 513 | 0.48 | [0.26; 0.88] | 7.5% | 5.4% |
| POPADAD, 2008 | 37 | 638 | 50 | 638 | 0.74 | [0.49; 1.12] | 12.0% | 11.6% |
| JPAD2, 2017 | 66 | 1262 | 73 | 1277 | 0.91 | [0.66; 1.26] | 17.4% | 18.6% |
| ASCEND, 2018 | 202 | 7740 | 229 | 7740 | 0.88 | [0.73; 1.06] | 55.0% | 56.1% |
| **Fixed effect model** | | **11454** | | **11468** | **0.84** | **[0.73; 0.97]** | **100.0%** | **--** |
| **Random effects model** | | | | | **0.84** | **[0.73; 0.97]** | **--** | **100.0%** |

Heterogeneity: $I^2 = 0\%$, $p = 0.65$

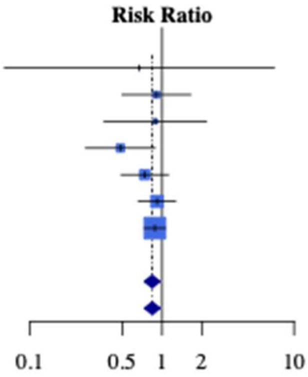


**Coronary heart disease**

| Study | Aspirin Event | Aspirin Size | Placebo Event | Placebo Size | RR | 95%−CI | Weight (fixed) | Weight (random) |
|---|---|---|---|---|---|---|---|---|
| TPT, 1998 | 4 | 29 | 6 | 39 | 0.90 | [0.28; 2.89] | 0.9% | 1.0% |
| HOT, 1998 | 21 | 752 | 27 | 749 | 0.77 | [0.44; 1.36] | 5.0% | 4.4% |
| POPADAD, 2008 | 90 | 638 | 82 | 638 | 1.10 | [0.83; 1.45] | 15.1% | 17.7% |
| JPAD2, 2017 | 63 | 1262 | 70 | 1277 | 0.91 | [0.65; 1.27] | 12.8% | 12.5% |
| ASCEND, 2018 | 279 | 7740 | 306 | 7740 | 0.91 | [0.78; 1.07] | 56.2% | 54.2% |
| ASPREE, 2018 | 54 | 1027 | 55 | 1030 | 0.98 | [0.68; 1.42] | 10.1% | 10.3% |
| **Fixed effect model** | | **11448** | | **11473** | **0.94** | **[0.84; 1.06]** | **100.0%** | **--** |
| **Random effects model** | | | | | **0.94** | **[0.84; 1.06]** | **--** | **100.0%** |

Heterogeneity: $I^2 = 0\%$, $p = 0.86$

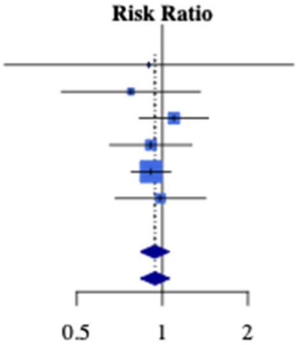


**Major Gastro-intestinal Hemorrhage**

| Study | Aspirin Event | Aspirin Size | Placebo Event | Placebo Size | RR | 95%−CI | Weight (fixed) | Weight (random) |
|---|---|---|---|---|---|---|---|---|
| PPP, 2003 | 8 | 519 | 1 | 512 | 7.89 | [0.99; 62.87] | 0.7% | 4.2% |
| POPADAD, 2008 | 28 | 638 | 31 | 638 | 0.90 | [0.55; 1.49] | 21.4% | 30.5% |
| JPAD2, 2017 | 25 | 1262 | 12 | 1277 | 2.11 | [1.06; 4.18] | 8.2% | 22.7% |
| ASCEND, 2018 | 137 | 7740 | 101 | 7740 | 1.36 | [1.05; 1.75] | 69.7% | 42.6% |
| **Fixed effect model** | | **10159** | | **10167** | **1.37** | **[1.11; 1.69]** | **100.0%** | **--** |
| **Random effects model** | | | | | **1.43** | **[0.92; 2.22]** | **--** | **100.0%** |

Heterogeneity: $I^2 = 57\%$, $p = 0.07$

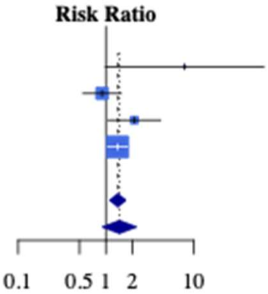

Major Intracranial Hemorrhage

| Study | Aspirin Event | Aspirin Size | Placebo Event | Placebo Size | RR | 95%-CI | Weight (fixed) | Weight (random) |
|---|---|---|---|---|---|---|---|---|
| PPP, 2003 | 0 | 519 | 0 | 512 | | | 0.0% | 0.0% |
| WHS, 2005 | 2 | 514 | 2 | 513 | 1.00 | [0.14; 7.06] | 3.1% | 3.0% |
| POPADAD, 2008 | 2 | 638 | 3 | 638 | 0.67 | [0.11; 3.98] | 4.6% | 3.6% |
| JPAD2, 2017 | 11 | 1262 | 15 | 1277 | 0.74 | [0.34; 1.61] | 23.0% | 19.1% |
| ASCEND, 2018 | 55 | 7740 | 45 | 7740 | 1.22 | [0.83; 1.81] | 69.3% | 74.3% |
| **Fixed effect model** | | **10673** | | **10680** | **1.08** | **[0.77; 1.51]** | **100.0%** | **--** |
| **Random effects model** | | | | | **1.08** | **[0.77; 1.52]** | **--** | **100.0%** |
| Heterogeneity: $I^2 = 0\%$, $p = 0.67$ | | | | | | | | |

All Caused Death

| Study | Aspirin Event | Aspirin Size | Placebo Event | Placebo Size | RR | 95%-CI | Weight (fixed) | Weight (random) |
|---|---|---|---|---|---|---|---|---|
| HOT, 1998 | 40 | 752 | 36 | 749 | 1.11 | [0.71; 1.72] | 3.5% | 3.5% |
| PPP, 2003 | 25 | 519 | 20 | 512 | 1.23 | [0.69; 2.19] | 1.9% | 2.0% |
| POPADAD, 2008 | 94 | 638 | 101 | 638 | 0.93 | [0.72; 1.21] | 9.7% | 10.1% |
| JPAD2, 2017 | 19 | 1262 | 21 | 1277 | 0.92 | [0.49; 1.69] | 2.0% | 1.8% |
| ASCEND, 2018 | 748 | 7740 | 792 | 7740 | 0.94 | [0.86; 1.04] | 76.3% | 75.3% |
| ASPREE, 2018 | 87 | 1027 | 68 | 1030 | 1.28 | [0.95; 1.74] | 6.5% | 7.3% |
| **Fixed effect model** | | **11938** | | **11946** | **0.98** | **[0.90; 1.06]** | **100.0%** | **--** |
| **Random effects model** | | | | | **0.97** | **[0.90; 1.06]** | **--** | **100.0%** |
| Heterogeneity: $I^2 = 0\%$, $p = 0.46$ | | | | | | | | |

Cancer Death

| Study | Aspirin Event | Aspirin Size | Placebo Event | Placebo Size | RR | 95%-CI | Weight (fixed) | Weight (random) |
|---|---|---|---|---|---|---|---|---|
| POPADAD, 2008 | 25 | 638 | 31 | 638 | 0.81 | [0.48; 1.35] | 8.2% | 20.9% |
| ASCEND, 2018 | 309 | 7740 | 315 | 7740 | 0.98 | [0.84; 1.14] | 83.3% | 53.5% |
| ASPREE, 2018 | 48 | 1027 | 32 | 1030 | 1.50 | [0.97; 2.33] | 8.5% | 25.6% |
| **Fixed effect model** | | **9405** | | **9408** | **1.01** | **[0.88; 1.16]** | **100.0%** | **--** |
| **Random effects model** | | | | | **1.05** | **[0.79; 1.40]** | **--** | **100.0%** |
| Heterogeneity: $I^2 = 50\%$, $p = 0.13$ | | | | | | | | |

Cardiovascular death

| Study | Aspirin Event | Aspirin Size | Placebo Event | Placebo Size | RR | 95%-CI | Weight (fixed) | Wei (rand |
|---|---|---|---|---|---|---|---|---|
| HOT, 1998 | 23 | 752 | 26 | 749 | 0.88 | [0.51; 1.53] | 13.2% | 13 |
| PPP, 2003 | 10 | 519 | 8 | 512 | 1.23 | [0.49; 3.10] | 4.1% | 4 |
| POPADAD, 2008 | 43 | 638 | 35 | 638 | 1.23 | [0.80; 1.89] | 17.8% | 21 |
| JPAD2, 2017 | 3 | 1262 | 6 | 1277 | 0.51 | [0.13; 2.02] | 3.0% | 2 |
| ASCEND, 2018 | 105 | 7740 | 122 | 7740 | 0.86 | [0.66; 1.12] | 61.9% | 59 |
| **Fixed effect model** | | **10911** | | **10916** | **0.93** | **[0.77; 1.14]** | **100.0%** | |
| **Random effects model** | | | | | **0.94** | **[0.77; 1.14]** | **--** | **100.** |
| Heterogeneity: $I^2 = 0\%$, $p = 0.55$ | | | | | | | | |

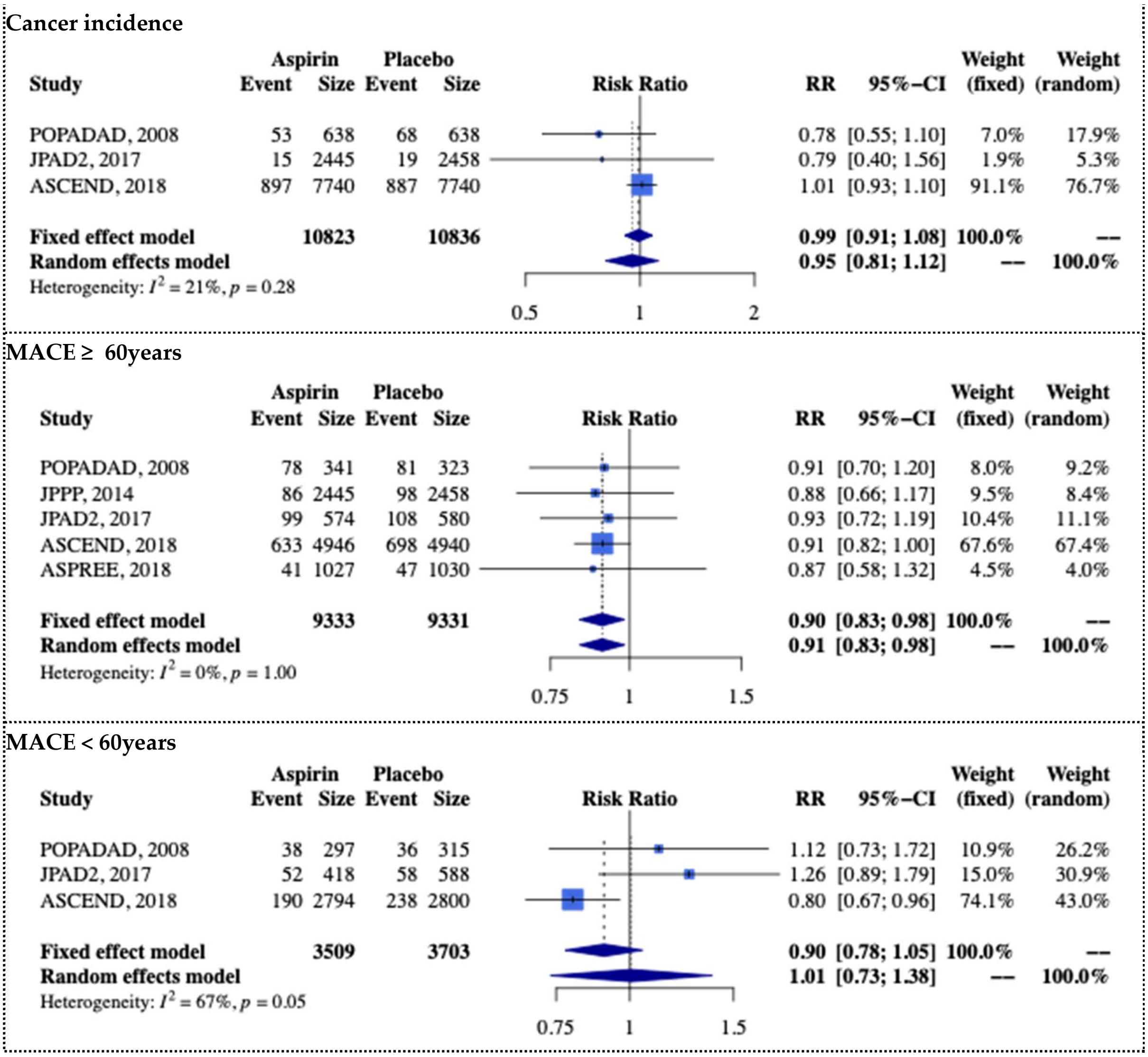


**Figure 2.** Forest plots of secondary outcomes, efficacy points and safety points in participants with diabetes for aspirin intervention trials.

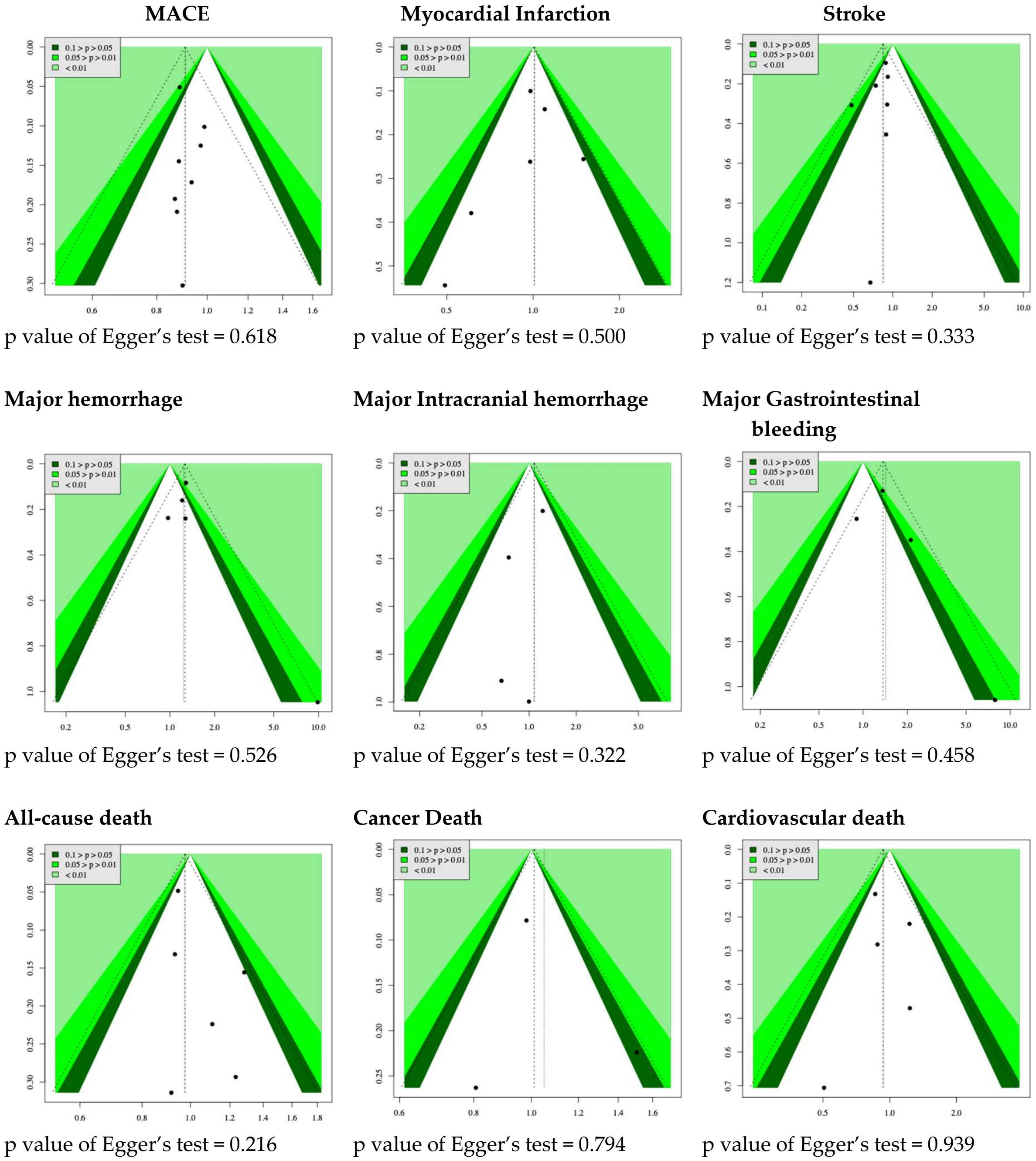


**Figure 3.** Funnel plots of major outcomes in participants with diabetes for aspirin intervention trials.
